# Causes of Phenotypic Variability and Disabilities after Prenatal Viral Infections

**DOI:** 10.3390/tropicalmed6020095

**Published:** 2021-06-01

**Authors:** Youssef A. Kousa, Reafa A. Hossain

**Affiliations:** 1 Division of Neurology, Children’s National Hospital, Washington, DC 20010, USA; 2 Department of Genomics and Precision Medicine, School of Medicine and Health Sciences, The George Washington University, Washington, DC 20052, USA; 3 Structural Virology Section, Laboratory of Infectious Diseases, National Institute of Allergy and Infectious Diseases, National Institute of Health, Bethesda, MD 20892, USA; hossaira@dukes.jmu.edu

**Keywords:** prenatal viral infections, Zika virus, flaviviruses, global child health, neurodevelopmental disabilities, brain development, phenotypic variability

## Abstract

Prenatal viral infection can lead to a spectrum of neurodevelopmental disabilities or fetal demise. These can include microencephaly, global developmental delay, intellectual disability, refractory epilepsy, deafness, retinal defects, and cortical-visual impairment. Each of these clinical conditions can occur on a semi-quantitative to continuous spectrum, from mild to severe disease, and often as a collective of phenotypes. Such serious outcomes result from viruses’ overlapping neuropathology and hosts’ common neuronal and gene regulatory response to infections. The etiology of variability in clinical outcomes is not yet clear, but it may be related to viral, host, vector, and/or environmental risk and protective factors that likely interact in multiple ways. In this perspective of the literature, we work toward understanding the causes of phenotypic variability after prenatal viral infections by highlighting key aspects of the viral lifecycle that can affect human disease, with special attention to the 2015 Zika pandemic. Therefore, this work offers important insights into how viral infections and environmental teratogens affect the prenatal brain, toward our ultimate goal of preventing neurodevelopmental disabilities.

## 1. Introduction

Prenatal infections are a leading cause of 2.6 million neonatal deaths globally each year. In addition, such infections increase the risk for preterm birth and contribute 11% of cerebral palsy risk [1,2,3,4]. In a series of 118 cases, prenatal infections were identified in 39% of second-trimester deaths and accounted for 45% of abortions [5].

Parental viral infections are of greatest concern because they are often difficult to diagnose, and the developing brain is uniquely susceptible to severe injury [6,7]. Of significant note, prenatal viral infections tend to cause the same types of neuropathology and neurodevelopmental disorders in a clinical continuum that can include microencephaly, global developmental delay, intellectual disability, refractory epilepsy, deafness, retinal defects, and cortical-visual impairment (see Table for selected prenatal viral infections and a summary of their associated features and clinical outcomes [3,4,5,8,9,10]). In fact, while some classic features are pathognomonic, prenatal viral infections are almost clinically indistinguishable by neuroradiographic criteria or clinical outcomes alone [10] Serious clinical outcomes like these result from the viruses’ overlapping neuropathology, on the one hand, and from the host’s common neuronal and gene regulatory response to infections, on the other [6].

Despite the epidemiologic and clinical significance of this problem, no prenatal standards of care in treatment are available today, nor can virally induced prenatal brain injury yet be targeted for prevention. This reality calls for a deeper understanding of the corresponding neurobiology, which remains extremely limited, as a critical step toward preventing and addressing prenatal brain injury.

As a case in point, during the 2015 Zika pandemic, prenatal viral infection was identified as the cause of congenital Zika syndrome (CZS), a clinical entity that can include severe microencephaly and a partially collapsed skull, among other specific features common to most prenatal viral infections (Table 1) [11]. In the past 5 years, prenatal Zika infection has been shown to cause a spectrum of neurodevelopmental disabilities [12,13]. Importantly, these features can occur in isolation or as a combination of phenotypes [12,13,14,15,16]. Studies are ongoing to determine all of the clinical and developmental manifestations of prenatal infection, but we already know that some infants can be severely or moderately affected while other infants appear to be completely unaffected at present.

**Figure 1:**
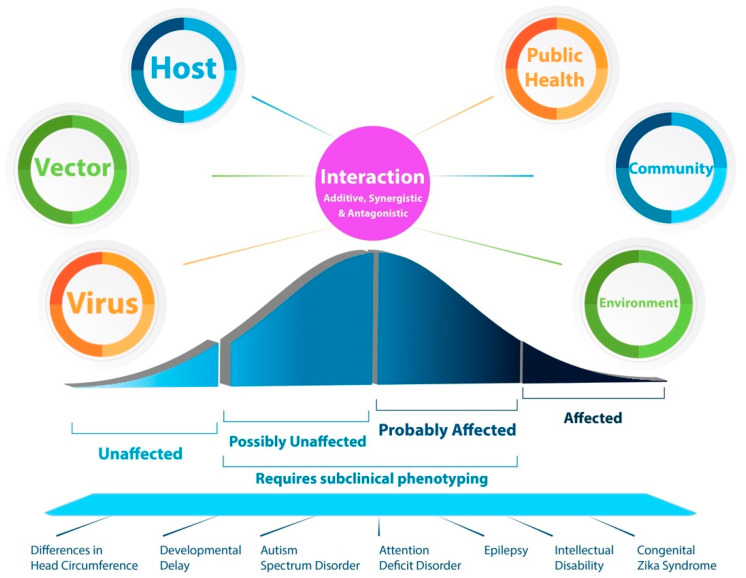
Interaction between possible disease modifiers and the spectrum of outcomes associated with prenatal viral infections. Various factors, including viral, vector, host, medical, community, and environment, may contribute to the spectrum of neurodevelopmental disabilities seen after prenatal viral infection, including in the case of Zika virus. These, in-turn, may interact in additive, synergistic, or antagonistic ways, which are yet unclear. The resulting phenotypic spectrum includes infants who are unaffected, possibly unaffected, probably affected, or affected. Affected individuals are commonly identified in appropriate clinical care settings, but the majority of individuals who are affected will likely require subclinical phenotyping, which can include neuroimaging, serial neurodevelopmental follow-up, and quantitative morphometrics. The resulting end-phenotypes can include a range of neurodevelopmental disabilities, which may include quantitative differences in head circumference, developmental attainment, the risk for seizures or epilepsy, social skills, attention, and intellect. Importantly, congenital Zika syndrome is but one manifestation of prenatal viral infection.

Variability in outcomes after prenatal viral infections suggests either a continuous phenotypic spectrum or discrete phenotypic clusters [7,11,12,13,14,15,17,18,19,20,21]. At this point, there is more evidence in support of discrete phenotypic clusters, but subtle quantitative effects may take longer to describe [12,13]. In either case, both models suggest quantitative phenotypic variability and differences in disease severity that may result from differences in the pathogen, vector, host, or environment or—more broadly—at any point in the virus–host cycle (Figure 1). (While a normal distribution is represented here given populational tendencies for quantitative characteristics (e.g., head circumference, height, weight, IQ), a nonparametric distribution is also possible. At this point, there is not sufficient data to know for sure which model is more accurate.)

To consider the causes of phenotypic variability after prenatal Zika virus infection and to gain a greater understanding of other prenatal viral infections, it is important to review the viral transmission lifecycle because, in theory, each of these stages (or variables) can have a downstream effect on the infected fetus. Validation of disease modifiers may point to therapeutically exploitable pathways for treatment or prevention of prenatal brain injury. In this work, we will review possible or likely factors that may contribute risk or protection in brain injury and the evidence where available. Overall, more studies are needed to identify and evaluate qualitative and quantitative disease modifiers and systematically deconstruct the causes of disease outcomes to identify those factors most amenable to public health or medical intervention.

## 2. Zika–Mosquito–Host Cycle

The Zika virus transmission cycle includes two parallel processes in nonhuman primates (Sylvatic) or humans (suburban–urban) that can interact or have crossover through the mosquito vector. In both cycles, the virus is carried to a host by a mosquito vector or by direct human to human transmission. This is followed by viremia and downstream effects on the host(s). Mosquitos take a blood meal from an infected host and are infected by the virus. The virus then sequesters in the salivary gland of the mosquito vector. During subsequent blood meals by the mosquito, viral particles are injected into the host from the mosquito’s salivary glands. The cycle continues after viral infection of another host. Differences in the lifecycle that affect viremia, neurotropism, or viral pathogenicity, likely contribute to the clinical outcome of the infected fetus.

**Figure 2:**
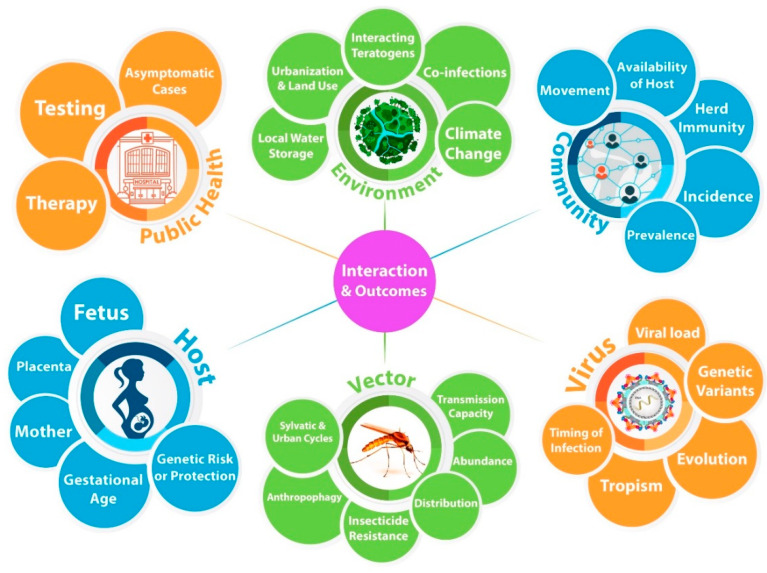
A model to consider possible modifiers of phenotypic variability in neurodevelopmental outcomes toward the long-term goal of prenatal precision therapies. There are likely primary drivers of neurodevelopmental disabilities. Moving clockwise, primary drivers include the environment, community, virus, vector, host, and public health efforts. Each primary driver is composed of highly interdependent factors. Epidemiologic, biomedical, and entomology research suggests the interaction among primary drivers can be additive, synergistic, or antagonistic. In multiple ways, the primary drivers are mutually interdependent in their outcomes. In order to develop personalized therapies to prevent neurodevelopmental disabilities, it is critically important to understand how (and when) these variables increase or decrease the risk of disease and to further delineate how they interact. In this way, personalized treatments or prevention strategies can be considered prenatally for viral infections, and other disorders affecting the developing brain.

The following sections cover differences in the virus, mosquito vector, human host, and their context, including public health and medical infrastructure, local community, and broader environment that can affect disease outcome after prenatal viral infection (Figure 2). Because of the number of people who were infected and are affected and mobilization by national and international research centers and funders to study this global health problem [22,23], the 2015 Zika virus pandemic offers a unique opportunity to study causes of phenotypic variability after prenatal viral infection and other environmental teratogens that affect the developing human brain.

## 3. Virus

Multiple streams of evidence strongly suggest that differences in the virus, including tissue tropism [24,25], viral strain [26], and evolution [27], contribute to phenotypic variability and disease severity in the infected fetus. First isolated from rhesus monkeys in the Zika forest of Uganda in 1947, Zika virus is phylogenetically categorized as a flavivirus [28]. This family of viruses includes many emerging human pathogens of clinical and public health significance, including Chikungunya, Dengue, yellow fever, and West Nile virus. Like other flavivirus, Zika virus has a small, single-stranded RNA genome, and it targets neural tissue [29] and non-neural tissue alike, including the placenta, testis, and uterus [4]. Unlike other flaviviruses, Zika can be transmitted between humans by exchange of bodily fluids and by sexual transmission [30,31,32,33]. In the postnatal brain, Zika leads to several infectious, para- and post-infectious, and inflammatory neurological disorders, affecting both the peripheral and central nervous systems and ranging from Guillain-Barre Syndrome to meningoencephalitis [34,35,36,37,38]. In mouse models, adapted viral strains result in similar developmental defects with prenatal [39,40,41,42,43,44,45,46,47,48,49,50,51,52] or postnatal infection [24,53].

## 4. Viral Evolution Likely Contributed to Pathogenicity

Mutations and evolution in the Zika viral genome are associated with increased viral pathogenicity. Sequence analyses have revealed three major genetic lineages of Zika virus; East African, West African, and Asian [26,54,55]. While genetically related, each viral strain evolves new characteristics that contribute to variable neuropathology. In one example, the nonstructural protein 1 (NS1) enhances viral prevalence in mosquitos and the mammalian population [54]. Analysis of the African lineages revealed a mutation at the 188th residue, from alanine to valine, which conferred secretability of the NS1 protein [54,56], and this may confer highly efficient enzootic and epizootic transmission [54,57,58]. Mutations in the nonstructural protein 3 (NS3) region, have been shown to increase viral replication in the mosquito, while decreasing replication in human cells [59]. Residues in the viral envelope (E) protein, including isoleucine 152, threonine 156, and histidine 158, have been implicated in the binding efficiency of Zika virus to host cells [60]. It is possible that viral strains co-evolved with their hosts and vectors, a theory that accounts for Zika’s long-term potentiation and re-emergence throughout the 20th and 21st centuries [54].

Zika’s unique ability to transfer directly between humans also suggests increased stability in bodily fluids, which may contribute to disease severity [25,61]. Cryogenic electron-microscopy of Zika incubated at different temperatures suggests high stability of viral particles at 40 °C in urine, saliva, or semen, in contrast to Dengue, which is not detected in these bodily fluids [61,62,63,64,65]. However, when studying the infectivity of Zika after incubation at 40 °C, the half-life (11.8 h) was comparable to Dengue (5.2 h) and West Nile virus (17.7 h), suggesting that viral pathogenicity is not only related to stability [62,66].

## 5. Mosquito

Flavivirus, including Zika, are transmitted to humans by Aedes mosquitoes, which have an expanding global distribution that now includes every continent and 41 of the 48 contiguous United States [28]. Zika virus is preferentially transmitted by some species of the *Aedes* mosquito, including *A. aegypti*, *A. albopictus*, *A. hensilli*, and *A. polynesiensis* [67] Similar to viral and host factors, differences in the Aedes mosquito can affect clinical outcomes [68] because they can affect the viral load that reaches the host. For instance, a recent study identified enhanced viral susceptibility in the evolution of a subspecies of *A. aegypti aegypti,* which invaded countries in the tropical belt. The change in the mosquito population was a major factor in the pathogenesis of Zika virus, and other flaviviruses [68] Combined with a preference to lay eggs in human-made containers [69], it appears that *A. aegypti aegypti’s* abundance in South America compared to Africa contributed to disease risk during the pandemic [68]. Conversely, African species of the mosquito, *A. ageypti formosus*, appears to be more resistant to Zika virus [68]. *A. aegypti aegypti* also has an allelic variation in the Or4 odorant receptor gene that is linked to a preference for biting humans [70,71]. It is possible that increased mosquito susceptibility results from genetic drift, adaptation to human urbanization, and/or region-specific factors, including climate change and human genetic changes [71].

In the western Pacific, such as the Federated States of Micronesia (FSM) and Palau, a 2007 outbreak of Zika virus was brought to the islands by a mosquito [72,73]. Introduction of the mosquito, along with the lack of herd immunity, contributed to the pandemic in this region. In addition, intense air and sea traffic could introduce mosquito species into different geographic regions across the globe [72,73]. The density and type of endogenous mosquito populations can affect the ability of novel mosquito species to establish new territory [72]. Artificial and natural habitats could support further evolution of various mosquito species [72], an approach that is considered in limiting mosquito populations that contribute to human disease [74].

## 6. Host

### 6.1. Gestational Age Confers Susceptible to Zika Pathogenesis

Epidemiological data from multiple countries strongly suggests that viral infection during the first trimester of gestation is associated with the greatest risk of severe neurodevelopmental disorders, including microcephaly [25,75] and other developmental abnormalities [76]. Consistently, Coutinho et al.’s prospective population-based study found a strong association between poor infant prognosis when mothers’ were infected with Zika virus during the first trimester (<12 weeks) of gestation [16]. These findings were corroborated by neuroimaging, which showed an eight times higher rate of brain abnormalities after first-trimester exposure compared to later in gestation. The risk of fetal anomalies was 14-fold higher [76].

### 6.2. Phenotypic Variation: From Congenital Zika Syndrome to Mild, and Subclinical Developmental Delay

In several recent studies, Zika-exposed children with normal clinical examinations and neuroimaging at birth went on to develop mild to moderate developmental delay and developmental disabilities [12,13,76]. Often, initially asymptotic infants had a developmental delay of 3–12 months of age. These findings included poor head growth or worsening developmental delay over time [77]. Prenatal Zika infection may also increase the risk for autism spectrum disorder [77,78]. Exposure to other viruses in the *Flaviviridae* family may also affect development, a factor that might be heightened when children are exposed to multiple related viruses [16,77,79]. Given this variability, physicians caring for patients with suspected exposure or those working in endemic areas should continue to closely evaluate head circumference and developmental attainment of prenatally exposed infants, even when infants are asymptomatic at birth [80]. A systematic approach is needed to assess the impact of Zika virus infection at specific gestational ages so that physicians, parents, and local resources can guide care and therapy for children with increased risk for delay or disability [16].

### 6.3. Host Genetic Modifiers after Prenatal Zika Infection

Powered by genetics and genomics, human susceptibility to infections is a subject of increasing interest over the past 60 years [81]. Among the first of these studies, a higher concordance rate of tuberculosis infection was identified in monozygotic twins compared to dizygotic twins [82], suggesting host genetic factors can contribute to increased susceptibility to infections. More recently, genetic susceptibility was identified for *Plasmodium falciparum*, a mosquito-borne unicellular protozoan that leads to malaria in childhood [83,84]; for HIV, a sexually transmitted RNA virus that can be congenitally acquired [10,85,86], and for Dengue, an emerging mosquito-borne flavivirus with a single-stranded RNA genome [87]. Zika is similar to these infectious agents because it is a mosquito-borne, prenatally acquired, and an RNA virus.

Interestingly, while many of these genetic modifiers confer risk [83,85], others factors confer protection [81,87,88,89]. The human genetic architecture of risk and protection is best characterized for HIV, where host genetic factors can affect viral load, disease progression, viral clearance, viral control, and chronic infection [85,88,89,90,91,92,93,94,95,96,97,98]. Mapping these genes illuminates critical genetic and cellular pathways that constitute the host response to viral infections and provides insights into creating preventative and therapeutic strategies and, ultimately, into producing rationale vaccine candidates.

Looking at it from the host’s vantage point, there are three [99] important Zika targets and thus potential host modifiers of prenatal brain injury: the mother [16,100,101], placenta [102], and fetus [103]. For example, identical twins have similar types of brain injury, whereas, fraternal twins have different levels of brain injury [103,104]. This suggests genetic susceptibility in the fetus can affect neurodevelopmental outcomes [53,104]. As an example, human neurodevelopmental protein Musashi 1 affects Zika replication [105], and differences in *mTOR* signaling correlate with phenotypic differences among discordant dizygotic twins [103]. In addition to genetic differences between infants, different human populations appear to be affected differently as well [7,18,55]. This further suggests that genetic differences between populations can also contribute to risk. 

In addition to neurodevelopmental gene expression, differences in host immune response, including T-cells, immunoglobulin, and mucin (TIM1) phosphatidylserine transmembrane receptors, have also been implicated in Zika-binding to a wider range of host tissues compared to Dengue [25,106]. TIM1 is also present in the human placenta, which might be a mechanism for placental infection, and could be a therapeutic target to prevent fetal infection [25,106]

### 6.4. Differences in Head Circumference after Prenatal Zika Infection Offers a Unique Opportunity to Study a Variable Clinical and Quantitative Phenotype with Impact on Human Development

Of the phenotypes associated with Zika virus, microcephaly and microencephaly were among the first to be recognized [107,108,109,110,111]. According to the CDC, microcephaly affects one in every 800 to 5000 infants and is defined as head circumference less than two standard deviations from the mean, for age and sex. In contrast, microencephaly refers to abnormalities in the brain tissue itself, defined as a brain weight two standard deviations below the mean. Microencephaly is an important medical problem to consider because it is associated with intractable epilepsy, cerebral palsy, global developmental delay, intellectual disability, and ophthalmologic disorders [112]. Studies on microencephaly are harder to perform because they often require neuroimaging, whereas microcephaly is evaluated routinely by measuring head circumference. Microcephaly and microencephaly often, but do not always, correlate directly.

Like many human conditions, microcephaly is genetically complex, which means both genetic and environmental factors contribute to risk [108]. Only 12 genetic loci are known in congenital microcephaly [113], but many more genetic syndromes or forms of brain injury can include microcephaly as a phenotypic component [114]. In addition to congenital presentations, microcephaly can also result from poor head growth over time (postnatal onset) [108,112]. The drivers of microencephaly include disorders in neurogenesis, neuronal cell death, or defects in neuronal maturation [108]. In addition to genetic causes, multiple environmental factors can result in microencephaly from brain injury (acquired), including malnutrition and other infections [10,108,115].

Interestingly, Zika is unique among infectious etiologies because it can lead to both congenital and postnatal onset microencephaly [116,117,118,119]. As a quantitative human trait, differences in head circumference can predict developmental attainment after Zika virus infection [12]. Therefore, the Zika pandemic offers a unique opportunity to study how environmental infectious pathogens, maternal-placental-fetal neuroimmune activation, and genetic modifiers affect postnatal human developmental attainment.

## 7. Public Health, Community, and Environmental Factors

### 7.1. Public Health and Medical Considerations

To date, as with other prenatal viral infections, it is difficult to diagnose prenatal Zika infection because the symptoms are nonspecific, if they occur [28,118]. Furthermore, there are no approved therapies to treat prenatal Zika virus infection or to prevent its associated developmental disabilities when (or if) an infection is detected [120,121]. Efforts are ongoing to identify and test vaccine candidates and therapeutic modalities [122]. When an infant is affected, a systematic and integrated approach is needed to assess the total impact relative to the gestational age at exposure so that parents and physicians can anticipate care for children with brain injury [16].

### 7.2. Impacts on Transmission

Investigations into the 2007 outbreak on the Yap Islands found immature forms of *Ae. aegypti*, *Ae. hensilli*, *Ae. lameliferus*, *Ae. maehleri*, and *Ae. vexans* in water-holding containers such as tires, tarp, water barrels, water tanks, and animal pans with the highest proportion in tires [72,123]. In the case of Brazil, standing water, improper disposal of garbage, and densely populated areas constitute potent breeding grounds for *Ae. Aegypti* [56,124]. A similar situation was seen with Dengue in Thailand, which likely contributed to the number of people affected and disease severity by impacting the overall viral load in the host [56,125,126]. Cumulatively, there is an increased risk of transmission, especially in urban populations where *Aedes* proliferation, and in turn viral load, is only mitigated by human behavior [55,56]. Other environmental factors include temperature, relative humidity, annual rainfall, and exposure to other teratogens.

### 7.3. Interventions to Combat Vector Density

It may be possible to reduce mosquito density by safely storing still water or disposing of artificial water containers, thus reducing mosquito access to breeding grounds. Pyriproxyfen can be used to prevent mosquito maturation into the adult form and allows predators to consume the larvae, overall decreasing the mosquito population [127]. Fumigation techniques and insecticides are important to consider, but their impact on the environment and pregnancy is unknown. Recent promising efforts have focused on using Wolbachia (a bacteria that infects mosquitos and blocks their viral infection) to reduce the transmission of mosquito-borne viruses [74].

### 7.4. Socioeconomic Factors as Drivers and Modifiers of Clinical Outcomes

Multiple studies have identified socioeconomic drivers for neurodevelopmental disabilities. For instance, higher maternal education reduced the risk of microcephaly prior to the pandemic in Brazil [128,129]. Further, lower household income and higher crowding was a risk factor for microcephaly [56,129]. Co-occurring health risks, such as malnutrition, increase the risk to the fetus [129]. Similar socioeconomic health modifiers should be considered and modeled in more developmental diseases [128]. Local efforts, for example, educating lower income households on Zika, can increase health-seeking behavior in socially marginalized groups and may decrease disease burden in future pandemics [129,130].

### 7.5. Global Climate Change

Global climate change may also increase the incidence of prenatal viral infection [131]. Previous modeling of *Aedes*-borne pathogens, such as Dengue, suggests that climate change increases the growth rate of *Aedes*, and in turn Zika virus, by lowering the outbreak threshold [124,132]. Temperature differences may produce a larger population of *Aedes* species, and increase exposure to potential virus-carrying vectors [132]. While considering various socioeconomic scenarios, approximately 1.3 billion new people are predicted to shift into geographic regions with favorable conditions for Zika and other viral infections [131]. Along with temperature changes, and the expected increase in vector density, there is a higher risk for local transmission in Europe and North America [132]. Though modeling is heavily dependent on multiple factors, these insights can be used to guide preliminary responses in case of a future outbreak [132].

## 8. A Conclusion and Perspective toward Understanding Phenotypic Variability after Prenatal Viral Infections

Causes of phenotypic variability after a prenatal viral infection are emerging but are as yet unclear (Figure 1). Furthermore, their relative contributions (and their interaction) to risk or protection in disease are unknown. Recent studies suggest that some of these factors can include differences in the virus, vector (mosquito), host (which includes the mother, placenta, and fetus), medical care (including early diagnosis and treatment), local community, and environment. Each of these factors might interact with in additive, synergistic, or antagonistic ways that affect the development of the central nervous system. Clinical and epidemiologic data shows a phenotypic spectrum of neurodevelopmental disabilities after prenatal viral infection. In fact, while some individuals are either unaffected or affected, the likely majority of individuals may be affected but require deep clinical (or subclinical) phenotyping over time to uncover the full impact based on recent studies [12,13]. Helpful phenotyping strategies can include neuroimaging, morphometrics, and long-term neurodevelopmental evaluations. These types of evaluations are often limited in low- and middle-income countries, which likely contributes to a systemic ascertainment bias for the number of affected individuals.

In turn, each discrete clinical problem associated with prenatal infection (microencephaly, developmental delay, autism, intellectual disability) than have a phenotypic spectrum of their own, which is either semi-quantitative or continuous. For example, an affected infant can have autism that is mild, moderate, or severe (semi-quantitative). Further still, each of these phenotypes may occur in isolation or in tandem or as part of a spectrum of disorders that result from prenatal brain injury.

Identifying these factors, and modeling their relative contributions, is critically important in understanding viral neurobiology in the parental brain and preventing neurodevelopmental disabilities. With this model in mind, it might be possible to identify pharmacologic therapies or risk mitigation strategies for pregnant women and their future infants. Such work could help us fully realize the long-term goal of prenatal precision medicine toward the prevention or treatment of neurodevelopmental disabilities (Figure 2), for Zika and beyond.

## Figures and Tables

**Table 1:** Prenatal viral infections lead to overlapping neuropathology and neurodevelopmental disabilities.

	CMV ^1^	HIV	HSV	LCMV ^2^	Rubella	VZV ^3^	Zika
**Calcifications**	✔	✔	✔	✔	✔		✔
**Cerebral Palsy/** **Motor Delay**	✔	✔	✔	✔	✔		✔
**Cerebellar Hypoplasia**	✔			✔			✔
**Chorioretinitis/Blindness**	✔	✔	✔	✔	✔	✔	✔
**Cortical Malformation**	✔		✔	✔		✔	✔
**Epilepsy/Seizures**	✔		✔	✔	✔	✔	✔
**Hearing Loss**	✔	✔			✔		✔
**Intellectual/** **Learning Disability**	✔	✔	✔	✔	✔	✔	✔
**Intraventricular** **Hemorrhage**						✔	✔
**Meningoencephalitis**	✔	✔	✔	✔	✔	✔	✔
**Microcephaly**	✔	✔	✔	✔	✔	✔	✔
**Myelination Disorder**		✔			✔		✔
**Neuropathies**							✔
**Vasculopathy/** **Porencephaly**		✔		✔	✔		✔
**Ventriculomegaly/** **Hydrocephalus**	✔		✔	✔			✔

^1^ Cytomegalovirus; ^2^ Lymphocytic Choriomeningitis Virus; ^3^ Varicella Zoster.
